# Life and experience as irreducible dimensions of nature

**DOI:** 10.3389/fpsyg.2026.1845620

**Published:** 2026-07-03

**Authors:** Fredric Schiffer

**Affiliations:** 1 Developmental Biopsychiatry Research Program, McLean Hospital, Belmont, MA, United States; 2 Department of Psychiatry, Harvard Medical School, Boston, MA, United States; 3 MindLight, LLC, Newton Highlands, MA, United States; 4 Private Practitioner, Newton Highlands, MA, United States

**Keywords:** biophotons, consciousness, dimensions, dual-brain psychology, force of experience, hard problem, irreducibility, life

## Abstract

Space and time are irreducible dimensions of nature—measurable but not derivable from anything more fundamental. Life and experience are here proposed as similarly irreducible dimensions. Together—sometimes referred to as lifeexperience to mark their inseparability—they constitute the dimensional ground from which emerges the Force of Experience—proposed as a candidate fifth fundamental force requiring further physical specification—whose causal reality is evidenced by the entirety of human civilization and all its products, for good and ill. Physics has failed to explain consciousness and life because it treats them as phenomena to be derived from lower-level processes, rather than recognizing them as foundational dimensions in their own right. Correcting this framing recasts both hard problems at once—not as derivation problems to be solved but as recognition problems to be addressed. Four converging arguments support these claims. First, the dimensional argument: Life and experience meet the same criteria for foundational status as space and time—they are causally real, irreducible, and known through inhabitation rather than external observation alone. Second, the AI argument: Information processing of extraordinary sophistication does not generate experience, providing an existence proof that something beyond computation is required. Third, the Now Problem: The present moment—the most certain datum of experience—has no place in the formalism of physics, a structural absence pointing to a dimension physics does not contain. Fourth, 33 years of clinical evidence: A single brain contains two selves independently accessing the experiential dimension, with therapeutic consequences for anxiety, depression, and opioid use disorder sufficient to earn FDA Breakthrough Device Designation. The Subjective Field is hypothesized as the physical mechanism linking the life dimension to the experiential dimension—transforming coherent living brain information into feelable information that can be received and actualized by the self. The field is physical and potentially discoverable; it takes brain information across a dimensional boundary. No physical instrument can yet follow this process. The hard problems are not solved by finding a better mechanism. They are reframed by recognizing that dimensions do not require mechanisms for their existence—they require only recognition of their irreducibility. The hard problems are addressed not by mechanism but by recognition—and from that recognition the hypothesized Subjective Field and Force of Experience, and the reported clinical evidence of two selves, take their proper place: not as derivations of consciousness from physics, but as features of a reality in which experience is foundational.

## Highlights

1. Life and experience are proposed as irreducible dimensions of nature alongside space and time.
2. The hard problems of consciousness and life are reframed as questions of recognition rather than derivation.
3. AI sophistication without experience proves computation is insufficient for consciousness.
4. The present moment—absent from physics—is direct evidence for the experiential dimension.
5. The Force of Experience, evidenced by all human civilization, is proposed as a fifth fundamental force.

## Section 1: the two hard problems

There are two problems in science that have resisted solution not because we lack data but because we have been asking the wrong questions. First is the hard problem of consciousness: Why do physical processes give rise to subjective experiences? The second, less often named but equally intractable, is the hard problem of life: Why does self-maintaining, goal-directed, meaningful organization exist, and why cannot physics derive it from chemistry? We clearly recognize that life exists, but how does it come about? Why does life only come from living organisms that reproduce to sustain life? Why is death, once established, irreversible even when most of the material is still present but not functioning?

Both problems have attracted serious effort for decades and curiosity for eons. The hard problem of consciousness was named by Chalmers (1996) and remains exactly as hard today as when he named it. The origin of life problem has occupied biologists and chemists for over a century. We know the chemistry of living systems with extraordinary precision—DNA, metabolism, homeostasis, signal transduction—yet we cannot explain how non-living chemistry crossed the threshold into a living entity. The persistence of both failures is curious. When smart people working hard with good data fail to solve a problem for decades, the problem may be framed incorrectly.

This paper proposes that both problems have indeed been framed incorrectly and in the same way. Both have been asked as questions of derivation: How does physics give rise to life, and how does life give rise to experience? Derivation is the right question when the phenomenon to be explained is a more complex arrangement of what came before. It is the wrong question when the phenomenon is genuinely new—a different kind of existence, not more of the same kind. I will argue that life and experience are genuinely new. They are not complex arrangements of physics. They are irreducible dimensions of nature, as fundamental as space and time, and the correct response to them is not derivation but recognition.

### The hard problem of consciousness

Chalmers drew a distinction that has proven durable. The easy problems of consciousness—how the brain discriminates stimuli, integrates information, directs attention, controls behavior—are discoverable by the methods of neuroscience and cognitive science. They are called easy not because they are simple but because they are the kind of problem those methods can in principle solve. The hard problem is different in kind. It asks why any of this processing is accompanied by subjective experience at all. Why does it feel like something to see red, to hear music, to be in pain? Why is there something it is like to be a conscious being, a feeling, an experience?

This question has not yielded to 30 years of serious effort. Neural correlates of consciousness have been mapped with increasing precision. Global workspace theory, integrated information theory, higher-order theories, predictive processing—each has illuminated something real about how the brain processes information. None has explained why that processing is accompanied by experience. Orch-OR has proposed that experience is produced by orchestrated quantum collapses but does not explain why such occurrences should be accompanied by consciousness. I will argue in Section 3 that this is not a contingent failure. It is a structural impossibility. You cannot reach the experiential dimension by going further within the physical dimensions, any more than you can reach the third dimension by traveling further within a plane, as in Abbott’s book, *Flatland* (Abbott, 1884).

### The hard problem of life

The second hard problem is less often named but has the same structure. Physics and chemistry describe the mechanisms of life with extraordinary detail. We know what living systems are made of and how they work. What we cannot explain is why a particular organization of matter crosses the threshold into goal-directed, self-maintaining, meaning-bearing existence—why it becomes alive.

The origin of life problem is not a problem of missing chemistry. It is a problem of missing categories. Something genuinely new enters at the threshold of life—not more complexity but a different kind of existence. A bacterium processing a chemical gradient is not merely a physical system responding to a stimulus. It is a system for which the gradient means something.

Approach or avoid. Toward food, away from acid. That meaning is real. It is not in the physics of the molecules. It belongs to a different dimension.

Schrödinger asked “what is life?” in 1944 (Schrödinger, 1944) and identified that living systems maintain local order against entropy in a way that physics alone does not fully explain. Prigogine formalized the thermodynamic conditions under which life-like organization can arise (Prigogine, 1977). Neither explained why some dissipative structures become goal-directed—why they acquire the property of being for something rather than merely existing. That property belongs to the life dimension. The cell or organism, when living, exists in a new domain, in a live dimension. The life dimension is like the dimension of time. Some physicists have suggested that time might not have existed prior to the big bang. But once the dimension of time exists, it can be measured and studied. Einstein showed that time will slow with speed and decreased gravity (Einstein, 1905, 1920). But time cannot be reduced to more fundamental properties. Neither can life.

### The same error underlies both

The hard problem of consciousness and the hard problem of life share a common structure. Both ask how a higher dimension derives from a lower one. Both assume that if we understand the lower dimension well enough, the higher one will follow. Neither assumption is correct. Space does not derive from anything more fundamental. Time does not derive from anything more fundamental. We do not explain space in terms of something else—we describe it, measure it, work with it, and accept it as foundational. The same is true of time. Both are irreducible dimensions of nature. We know them by inhabiting them, not by deriving them.

Life and experience meet the same criteria for foundational status. They are causally real—living systems do things non-living systems cannot, and experience demonstrably influences the brain and behavior. They are irreducible—no derivation has succeeded despite sustained effort.

They are known through inhabitation as well as external observation—you inhabit experience as first-person experience and you observe it as third-person experience.

The hard problems are hard because they have been asked as questions of derivation when they are questions of recognition. This paper does not propose another mechanism by which physics might give rise to experience. It establishes that the question has been wrongly posed—and that correcting the question recasts both problems at once.

## Section 2: what irreducibility means—the dimensional argument

What does it mean to say that something is irreducible? In science, we are accustomed to explaining phenomena by reducing them to something more fundamental. Water is explained by hydrogen and oxygen. Temperature is explained by heat and molecular motion. Heredity has been explained by DNA. These reductions work because water, temperature, and heredity are complex arrangements of simpler things. The explanation by reduction reveals what they are made of and how they work.

But some things are not complex arrangements of simpler things. Space is not made of anything more fundamental. Time is not made of anything more fundamental. We can describe space—measure its geometry, discover that it curves in the presence of mass, unify it with time in the spacetime of general relativity. We cannot explain what space is in terms of something else. It is foundational. The same is true of time. We can measure temporal intervals, describe their direction, show that time slows with speed and weaker gravity. We cannot reduce time to something more basic. Augustine (1991) asked “what is time?” in the fourth century. The question remains unanswered at the fundamental level. We use time. We do not explain it.

This is not a failure. It is the correct recognition that space and time are dimensions—foundational constituents of reality where explanation stops and description begins. Physics did not fail to explain space and time. Physics recognized them as foundational and built everything else on top of them. That recognition was the beginning of real physics, not the end of it. Space and time remain irreducible at all scales of physics. In relativity, they are radically altered—contracted, dilated, curved by mass–energy, stripped of absolute simultaneity—yet they are not reducible to anything more fundamental. In quantum regimes, they may disappear or change character at the smallest scales, but again, they are not reducible to anything else. Alteration under extreme conditions is not derivation. The dimensional framework here addresses life and experience as they occur at the ordinary scales of biology and consciousness, where space and time are unambiguously foundational.

### Knowing dimensions by inhabiting them

There is something distinctive about how we know dimensions. We do not know space by observing it from outside. We know it by inhabiting it—by touching objects, walking around them, seeing them from different angles. A being confined to two dimensions could not deduce the existence of a third dimension from within its plane, however intelligent it might be. Edwin Abbott explored this in his 1884 novel Flatland (Abbott, 1884). The two-dimensional Square encounters a three-dimensional Sphere but can only perceive it as a circle—a cross-section of something whose full nature is categorically inaccessible from within the plane. The Square does not lack intelligence. It lacks access. No amount of motion within the plane reaches the third dimension. The third dimension must be encountered, not derived.

This is precisely the situation in consciousness science. Every theory of consciousness has attempted to reach the experiential dimension by going further within the physical dimensions—more integration, more complexity, more self-reference, more information processing. None of them work. This is not because they are wrong about information processing. They are largely right about information processing. They are wrong about what information processing can produce. You cannot reach a new dimension by going further in the existing ones. The Square cannot find depth by exploring length and width more thoroughly.

We know the experiential dimension the same way we know space—by inhabiting it. You cannot observe experience from the outside. You can only have it. Every attempt to explain experience in third-person terms is an attempt to describe the interior of a dimension from its exterior. It produces correlates, not causes. Neural correlates of consciousness are the two-dimensional cross-sections of something whose full nature requires inhabiting the experiential dimension. They are real and worth mapping. They are not the thing itself.

### The test for dimensional status

How do we know when something deserves to be recognized as a dimension rather than explained as a phenomenon? I propose three criteria.

First, it must be causally real—it must make a genuine difference to what happens in the world. Space is causally real. Objects move through it, collide within it, and curve it with their mass. Time is causally real. Events succeed one another within it; causes precede effects. Life is causally real—living systems do things that non-living systems of equivalent chemistry cannot do. Experience is causally real—as I will show from 33 years of clinical evidence, felt experience demonstrably influences brain activity and behavior.

Second, it must be irreducible—it cannot be derived from or fully explained in terms of other dimensions. Space cannot be derived from time or matter. Time cannot be derived from space or energy. Life has resisted derivation from chemistry despite a century of effort. Experience has resisted derivation from neural activity despite 30 years of intensive work.

Third, it is known through inhabitation rather than external observation alone. We know space by being in it. We know time by living through it. We know experience by having it. This third criterion is what makes the experiential dimension unique among the four—it is the only dimension that is exclusively known from the inside. You can only correlate its physical behaviors and empathize with another’s distress, but you cannot experience it directly.

A skeptic may object that experience cannot be foundational because it cannot be measured from outside. But this objection rests on a hidden assumption that does not survive examination. Space cannot be measured from outside either. Neither can time. A being with no spatial extension could not measure space; a ruler requires a being already inhabiting space, comparing one extent to another. A being with no temporal duration could not measure time; a clock requires a being already inhabiting time, comparing one duration to another. Measurement is always done from inside the dimension by a being for whom that dimension is given. We do not measure space from some hypothetical outside; we measure it because we are already in it. The same is true of the experiential dimension. A being who inhabits it can measure what passes within it—by introspection, by phenomenological report, by inside observations of thoughts and feelings. The 0–10 anxiety scale routinely used in my clinical practice is a measurement of the experiential dimension from inside, by the being who inhabits it. It is no less a measurement than a ruler measuring a table. The demand that the experiential dimension be measurable from outside is not a neutral methodological standard. It is a category error that, applied consistently, would dissolve space and time as well.

Life and experience meet all three criteria. They deserve foundational status.

Ontic structural realism in philosophy of physics, developed by Ladyman, Ross, and French, provides a precise and rigorous basis for this dimensional analogy (Ladyman et al., 2007). What makes space and time foundational is not their intrinsic nature—which remains deeply contested in physics—but their structural necessity: Nothing in physics can be described without reference to a spatiotemporal framework. Life and experience are foundational in the same sense—not because their intrinsic mechanisms are fully understood, but because they are necessary conditions for their respective domains of description. Remove the life dimension and biological phenomena reduce to chemistry—goal-directedness, self-maintenance, and meaning disappear. Remove the experiential dimension and conscious phenomena reduce to information processing—the Now, feltness, and within-ness disappear. Temperature, by contrast, is not foundational in this sense—physical description does not require it as a necessary framework. Experience cannot be specified within the physical structural framework at all. Recognition of dimensional necessity precedes and does not require complete mechanistic derivation—which is precisely the recognition versus derivation argument the present framework advances.

As Descartes (1996) famously articulated, I know that I have experiences and I know that my experiences affect me, my brain, my feelings, and my thoughts (Schiffer, 2026a). How I enter this dimension will be discussed in Section 8, but I can point out here that I need a living, functioning brain for access. Once in the experiential dimension, I can know that I am in three dimensional space and that time passes. I do not know how I gained access to the dimensions of space and time, and I do not know how I entered the life dimension, but I know that that required access via information and structures passed on to me from my parents at conception (Walker and Davies, 2013).

### A universe without experience

Consider a universe without life—a universe of pure physics. Space exists. Time exists. Matter moves through spacetime according to physical laws. Information propagates. The universe expands or contracts, black holes exist, masses attract each other through gravity and may collide. Atoms interact through electromagnetic forces and are held together by the strong and weak nuclear forces. Causation operates. But nothing is experienced; nothing is known, nothing is observed. The block universe sits complete—all events coexist in the four-dimensional manifold, but no event is present, no moment is now, because “now” requires experience, it requires awareness.

The physicist’s universe confirms this absence. As I will discuss in Section 5, both Einstein and Gödel concluded that the present moment has no place in the physics of spacetime. The block universe contains all events coexisting with equal status. Nothing in physics singles out any moment as present. The now—the most certain datum of experience—is entirely absent from the formalism. This is not incidental. It is structural evidence that the now belongs to a different dimension.

Proper time, time in physics, is geometric, measuring the length of a spacetime trajectory. Entropy gives directionality—an arrow from past to future—but not presence. A universe with irreversible processes but no experiencing beings has a before and after, but no now. Things would happen in sequence. Nothing would be happening now because now is an experience, and only an experience.

I will return to this in Section 5, where I treat the now as the most direct physical evidence for the experiential dimension. For the moment, the point is simpler: The now exists only within the experiential dimension. It is one of the four properties that experience adds to the physical world—alongside for-ness, within-ness, and stakes. Remove the experiential dimension, and the universe has only physical, visionless interactions without aims, purpose, or contemplative meanings.

This means space and time, though they exist without experience, acquire meaning only through experience. They become known, measured, traversed, and meaningful only when an experiencing being inhabits them. The experiential dimension is therefore not merely one more thing in spacetime. It is the condition under which spacetime becomes a world rather than an unknown geometry.

### The ontological table

The relationship between the four dimensions can be stated precisely:

Space and time exist without life but are actualized—known, measured, traversed—only through the experiential dimension. The life dimension does not exist without specific biological conditions. The experiential dimension does not exist without the life dimension. It is doubly dependent—on life for its existence and on a receiving agential self for its full actualization.

The architecture is therefore: *Physical dimensions → [Life threshold].*

*→ Life dimension → [Experiential threshold] → Experiential dimension.*

Each threshold is a genuine phase transition—not a smooth increase in complexity but a categorical change in the kind of existence. Not all life crosses the experiential threshold. Bacteria and plants inhabit the life dimension—they process meaning-bearing information and have organism-relative responses. Whether they couple with the experiential dimension remains an open question. Higher neural systems with sufficient coherence and organism-relativity cross the second threshold. The mechanism of that crossing is the subject of Sections 7, 8.

The key point here is structural: The thresholds are real, the dimensions are irreducible, and the direction of explanation runs from recognition downward rather than from derivation upward. We do not explain experience by finding the right combination of physical processes. We recognize experience as a dimension and ask what physical conditions allow access to it.

### The correct ontology

The hard problems are hard because they have been asked as questions of derivation when they are questions of recognition. The correct questions are not how physics produces life and experience but what conditions allow access to each dimension—tractable questions that the following sections address.

## Section 3: why existing theories cannot cross the threshold

The hard problem of consciousness has attracted serious theoretical attention for 30 years. The theories that have emerged are serious efforts—each captures something real about how the brain processes information. But none of them explains why that processing is accompanied by experience. I will argue that this is not a contingent failure—not a matter of needing better data or more sophisticated models. It is a structural impossibility. Every existing theory attempts to reach the experiential dimension by going further within the physical dimensions. That cannot work. You cannot reach a new dimension by traveling further in the existing ones.

### Neural complexity and emergence

The most intuitive proposal is that consciousness emerges from neural complexity—that when information processing reaches sufficient sophistication, experience arises. Mallatt and colleagues have developed this position carefully (Feinberg and Mallatt, 2016), and Tononi’s integrated information theory represents its most mathematically rigorous form (Tononi, 2004). But complexity does not explain the transformation. Complex computers do not have experiences. A resting awake brain is less computationally complex than one solving a difficult mathematical problem, yet both are conscious. More fundamentally, no account of complexity explains why any physical process should feel like anything at all. Complexity is a property of organization within the physical dimensions. Experience belongs to a different dimension entirely. No amount of organizational complexity crosses that threshold.

### Global workspace theory, integrated information theory, higher-order theories, and predictive processing

Global workspace theory (Baars, 1988; Dehaene and Changeux, 2011) identifies the neural architecture of conscious access but does not explain why broad broadcast is accompanied by felt experience. Integrated information theory (Tononi, 2004) proposes that consciousness corresponds to *Φ*—a measure of integrated information—but Φ describes a property of organization, not why that organization feels like anything. To say that high Φ is consciousness is to name a correlation and call it an explanation. Higher-order theories (Rosenthal, 1986) propose that a mental state becomes conscious when represented as such by a higher-order state—but self-representation is itself information processing, and the hard problem is pushed back one level without being resolved. Predictive processing (Friston, 2010) is a powerful account of much that the brain does, but prediction error minimization can be implemented in silicon; if it were sufficient for experience, every thermostat would have a felt present moment. Each of these theories correctly describes something about how the brain processes information. None explains why any processing is accompanied by experience.

Predictive processing deserves extended engagement because it is the most empirically powerful current framework. Predictive processing, developed by Clark (2016), Friston (2010), and Seth (2021), proposes that the brain is a prediction machine that minimizes the gap between its internal model of the world and incoming sensory input. This framework has generated powerful empirical predictions across perception, attention, learning, and psychiatric disorders and represents the most sophisticated current neuroscientific account of the relationship between brain processes and experiential content. The present framework acknowledges the empirical power of predictive processing and regards it as compatible with the dimensional framework at the level of computational mechanism—predictive processing describes how the brain generates and refines the syntactical information that the Subjective Field then transforms into feelable information.

However, predictive processing does not address the hard problem of consciousness. As Seth explicitly acknowledges, the framework explains the structure and content of conscious experience but not the existence of experience itself (Seth, 2021). Why does minimizing prediction error feel like anything? Predictive processing has no answer. The dimensional framework addresses precisely this question—not by deriving experience from prediction error minimization but by recognizing the experiential dimension as foundational and identifying the Subjective Field as the physical transducer at the dimensional boundary.

Beyond this foundational limitation, predictive processing is built on the model of a rational Bayesian agent optimally updating its world model in response to prediction error. Clinical reality reveals this model to be profoundly and systematically limited in scope. Current WHO data reports that approximately 1 in 8 people worldwide—nearly 1 billion—are living with a mental health disorder at any given moment (World Health Organization, 2022). A Harvard-led study found that 50% of people will develop at least one mental health disorder over their lifetime by age 75 (Kessler et al., 2007). Neither figure includes the ordinary neurotic functioning—defensive, distorted, and irrational mental processing—that characterizes the majority of human psychology below the threshold of diagnosable disorder. The rational Bayesian updater that predictive processing describes applies to a small minority of human minds in a small minority of their moments. As a theory of how minds generally work, predictive processing describes the exception and treats it as the rule.

More fundamentally, predictive processing locates distortion at the level of attention and interpretation. Clinical observation reveals that distortion occurs at a deeper level—at perception itself. In published clinical trials and case reports spanning three decades, patients were shown a symmetrical photograph—constructed by taking one half of an enraged face, mirroring it horizontally, and combining the two identical halves into a perfectly symmetrical image (Schiffer et al., 2004, 2020; Schiffer, 2021). When viewed through different lateral visual fields, the same symmetrical stimulus was perceived entirely differently—through one visual field the face appeared genuinely frightening; through the other the identical face appeared silly, almost clownlike. Because the image is perfectly symmetrical, no difference in attended features can account for this perceptual difference. The entire difference is generated by the hemispheric self-structure through which the identical stimulus is processed. A predictive processing theorist might respond that two hemispheres with different developmental histories implement different priors over threat stimuli, producing different perceptual outputs from the same ambiguous image. We acknowledge this as a coherent computational description of how each hemisphere processes brain information. The dimensional framework operates at a level predictive processing does not reach. Predictive processing describes computational mechanisms—prediction, error minimization, prior updating—that operate on syntactical brain information. The same mechanisms are implemented by contemporary AI systems, which show no evidence of consciousness under any reasonable criterion. Predictive processing as currently implemented thus describes how brain information is generated and refined without producing evident conscious experience; it does not, on its own, explain how any processing becomes conscious experience. The dimensional framework specifies this transition: Brain information is transformed by the Subjective Field into feelable information, and reception of feelable information by a self constitutes entry into the experiential dimension, which is what consciousness is. The symmetrical photograph illustrates that two hemispheric selves with different developmental histories receive feelable information differently—but more fundamentally, it illustrates the existence of two distinct hemispheric selves, each capable of receiving feelable information.

Furthermore, predictive processing accounts of hallucinations and delusions treat them as failures of prediction error minimization—the brain’s internal model overriding sensory input. While this describes the computational mechanism, it does not account for the meaningful and purposeful content of hallucinations and delusions. Clinical observation and psychodynamic theory suggest that hallucinations and delusions are purposeful productions of a troubled brain hemisphere or unconscious brain information, functioning as unconscious defenses whose specific content reflects the patient’s psychological history and conflicts. The Force of Experience—the mechanism by which the specific meaningful character of experiential content acts causally on the brain and motivates action in the world—is not addressed by predictive processing. Friston, Seth, and Clark describe how prediction error drives action through active inference, but this account is syntactical—it explains why an organism acts to reduce discrepancy between model and world, not why pain compels avoidance, why love compels approach, or why meaning motivates creation. The causal role of experiential content as such—its specific felt character rather than its informational structure—is outside the predictive processing account (Schiffer, 2025).

### Orchestrated objective reduction

Penrose and Hameroff have proposed that consciousness arises from quantum mechanical processes—specifically, objective reduction of quantum superpositions in microtubules within neurons (Hameroff and Penrose, 1996). This is a serious proposal that at least gestures toward physics beyond classical computation.

But Orch-OR does not explain why quantum collapse should feel like anything. Quantum events occur throughout nature—in every atom, in every photon interaction. Inanimate quantum processes are not conscious. The proposal identifies a physical event as the candidate but does not explain the transformation from physical event to felt experience.

### Panpsychism

Chalmers, Goff, and others have proposed that experience is a fundamental property of all matter—that even elementary particles have some form of proto-experience, which combines upward into the rich experience of conscious beings (Chalmers, 2015; Goff, 2017). This has the virtue of taking experience seriously as a fundamental feature of reality rather than trying to derive it from something else.

But panpsychism distributes experience too broadly. Experience is not everywhere—it is biologically confined. Rocks do not experience. Stars do not experience. The Higgs field is omnipresent yet the photon remains massless because it lacks the coupling parameter to interact with it. Similarly, the experiential dimension may be a fundamental feature of reality without interacting with all matter. Biological confinement is the key: only living neural systems with sufficient coherence couple with the experiential dimension. Panpsychism also faces the combination problem—how do micro-experiences combine into the unified experience of a conscious self? That problem does not arise in my framework because experience is not assembled from smaller experiences. Instead, it is transformed from biological information by a field.

The predecessor frameworks that most directly anticipate the dimensional proposal deserve careful engagement. Chalmers’ naturalistic dualism establishes that phenomenal properties cannot be derived from physical description and must be treated as fundamental (Chalmers, 1996)—a conclusion the present framework endorses. However, naturalistic dualism is not essentially different from Cartesian dualism except in name—both assert that awareness and physical substance are ontologically distinct without providing an adequate account of their interaction. Descartes identified the thinking subject as the one indubitable fact and posited mind and body as distinct substances; Chalmers makes the same structural move without invoking God, but the interaction problem remains equally unresolved. Critically, neither framework requires life as a necessary condition for experience. The present framework differs in that experience is observed to be confined to living biological systems—not because this confinement is derived from first principles, but because the coupling condition is only met in living neural systems. This is an empirical observation treated as foundational, in the same way that the existence of spatial and temporal dimensions is treated as foundational rather than derived. The dimensional framework advances beyond naturalistic dualism in four specific ways: first, by proposing the life dimension as a necessary intermediate layer; second, by identifying the Subjective Field as a physical transducer that transforms syntactical brain information into feelable information; third, by proposing the Force of Experience as the mechanism by which the experiential dimension acts back on the physical world; and fourth, by grounding all three proposals in clinical evidence demonstrating that experiential access is biologically variable and selectively modulable through hemispheric stimulation.

Russellian Monism, developed by Goff, Strawson, and Chalmers, proposes that phenomenal properties are the intrinsic nature of physical properties—that what physics describes structurally from the outside is, from the inside, experiential in character. This framework correctly identifies that physics describes only structure and relations. The present framework shares the commitment to experience as ontologically fundamental. However, Russellian Monism leads naturally to panpsychism—distributing proto-experiential properties across all matter—and faces the unsolved combination problem. Both difficulties are avoided by a simpler and more empirically grounded observation: All experience originates in brain information. When I fall in love, my experience concerns a real relationship received through my sensory system and processed by my brain. When I am terrified, my brain has calculated that danger exists. There are no experiences without proximal brain information processed by a living neural system. Proto-experience in electrons is not required and not observed. Furthermore, Strawson argues that experience is more certain than any physical theory (Strawson, 2006). While the present framework agrees that experience is ontologically fundamental, Strawson’s claim overstates the certainty of experience in a clinically important way. Hallucinations, delusions, drug-induced states, and irrational fears demonstrate that experiential content can be systematically distorted. In every case, the character and content of experience are determined by brain information—hallucinations arise from purposeful productions of a troubled brain hemisphere functioning as unconscious defenses, not from random noise. This tight dependence of experiential content on specific brain information is inexplicable on the panpsychist account and entirely natural on the dimensional framework.

### The common structure of failure

Each of these theories describes something real about information processing in the brain. Global workspace theory correctly identifies the neural architecture of conscious access. Integrated information theory correctly identifies that integration matters. Predictive processing correctly describes much of what the brain does. These are genuine contributions to neuroscience, but they all fail to explain consciousness for the same reason. They are all working within the physical dimensions—describing how information is organized, integrated, broadcast, predicted, and represented. The experiential dimension is orthogonal to the dimensions they are working in. You cannot reach it by going further in the directions they are traveling.

Each theory describes the house. None of them explains the light inside it.

The next three sections present the converging evidence that something beyond information processing is required—and identify what that something is.

## Section 4: the AI argument—an existence proof

Before presenting the AI argument, the present framework requires explicit categorical definitions of experience and consciousness, terms often used interchangeably in the philosophical literature but that refer to distinct phenomena within the dimensional framework.

Experience is the broader category, encompassing all information processing that occurs within the experiential dimension—both non-conscious and conscious. We offer a speculative account of the consciousness threshold—a candidate mechanism whose refinement will require further theoretical and empirical work. We suggest both non-conscious and conscious experience require the transformation of syntactical brain information into feelable information by the Subjective Field. What distinguishes conscious from non-conscious experience is the further step of acceptance by the self. Feelable information competes within the self for entry into a global workspace. Each self determines what is accepted and actualized as the felt present conscious moment; feelable information not selected remains non-conscious. The two hemispheric selves may select different contents from the available feelable information, which accounts for the qualitatively different conscious states observed when one or the other self is dominant. Non-conscious experience includes biological information processing that has been transformed by the Subjective Field into feelable information but has not yet been actualized as conscious experience. Such non-conscious experience likely retains genuine experiential quality—an unconscious terror likely has an unconscious feeling to it—because it has already been transformed by the Subjective Field and therefore has partial access to the experiential dimension.

Consciousness is the narrower category—experience that has been actualized as the felt present conscious moment. All consciousness is experience but not all experience is conscious. Every conscious experience has three inseparable features present simultaneously: phenomenal character (there is something it is like to have it, in Nagel’s sense, Nagel, 2012); specific content shaped by the particular hemispheric self-structure accessing the experiential dimension; and force—it acts on the brain and through it on the physical world. These are not three inconsistent uses of the term experience. They are three inseparable and universal features of every conscious experience within the dimensional framework. Phenomenal consciousness refers to the qualitative character of experience. Access consciousness refers to information being available for use in reasoning and behavioral control—a functional rather than qualitative notion. Sentience refers to the capacity for felt sensation, particularly pain and pleasure.

Experiential dimensionality in the present framework refers to the foundational ontological status of experience as an irreducible dimension of nature, accessible through the biological coupling condition, and encompassing phenomenal consciousness, access consciousness, and sentience as manifestations of experiential access at different levels of biological complexity.

The argument of the previous sections can be stated simply: Experience belongs to a dimension that cannot be reached by going further within the physical dimensions. The strongest evidence for this claim comes not from philosophy but from the most significant technological development of our time. Artificial intelligence has become extraordinarily sophisticated. It has not become conscious. That gap is not incidental. It is diagnostic.

### What AI can do

Modern AI systems process information at a scale and sophistication that exceeds human performance across a remarkable range of cognitive tasks. They integrate information across vast contexts. They model their own uncertainty. They generate text, images, music, and code that is indistinguishable from human output in many domains. They engage in what appears to be reasoning, planning, and self-reflection.

### What AI cannot do

AI systems do not feel anything: not pain, not the pleasure of solving a problem, not the sound of music or the experience of color. There is no now for an AI system—no present moment from which the past is remembered and the future anticipated. There is processing and there is output. There is no experience of doing it.

This is not a speculation about hidden inner states; it is a structural fact about what AI systems are. They operate entirely within the physical dimensions—computation, information, pattern, causal structure. They have no access to the life dimension. They are not alive. They were not born. They do not maintain themselves against entropy. They do not have organism-relative responses to their environment in the biological sense. They process inputs and generate outputs according to learned statistical patterns.

Without access to the life dimension, there is no access to the experiential dimension. The architecture simply is not there.

### The existence proof

This gives us something precise: An existence proof that information processing, regardless of its complexity or sophistication, is insufficient for experience.

The argument is clean. AI processes information with extraordinary complexity. AI does not feel. Therefore, complexity and information processing are insufficient for experience. Therefore, something beyond information processing is required for experience. That something is access to the experiential dimension—and the life dimension that is its necessary gateway.

This is not a hypothesis. It is a logical consequence of two observations not seriously disputed: AI is sophisticated, and AI does not feel. The gap between them is not a matter of degree; it is a matter of dimension.

A functionalist will object that sufficiently complex computation might yet produce experience. This is the emergentist position—invoking complexity as the mechanism of experience without specifying what complexity does or at what threshold it acts. This is not an explanation; it is a placeholder. The coupling condition the dimensional framework specifies—coherent, temporally and spatially organized biological emissions meeting a biological threshold—is a principled criterion, not an appeal to complexity. Current AI does not meet this criterion because it is not a biological organism. Whether silicon could ever instantiate an equivalent but different form of life—meeting an analog of the biological coupling condition through a different physical substrate—is an open question the framework can acknowledge without conceding the main argument. What is clear is that current AI, however sophisticated, does not meet the coupling condition and therefore has no access to the experiential dimension at any level, conscious or non-conscious.

### What the gap reveals

The four properties of experience—for-ness, within-ness, now-ness, and stakes—are all absent in AI systems.

There is no for-ness: Nothing is for the AI system in a felt sense. Optimization targets and loss functions are imposed from outside. No information matters to the system from within.

*There is no within-ness*: There is no point of view that the processing inhabits. There is a model, but no experiencer.

*There is no now*: The system processes tokens in sequence but has no present moment. There is no here, no now, no felt passage of time.

*There are no stakes*: The system has no pain, no fear, no hunger, no grief, no urge. Nothing demands a response from within the information itself. External penalties can be imposed, but intrinsic stakes cannot be computed.

These are not features that could be added by scaling the model or improving the architecture. They are properties of a different dimension. A system that does not inhabit the life dimension cannot inhabit the experiential dimension. No amount of additional computation changes this.

### The question this raises

If information processing is insufficient for experience, what is sufficient? Something must transform syntactic information—neural information patterns with causal structure—into feelable information—patterns with the potential to be experienced. That transformation is real. We know this because experience exists and computation does not account for it.

What produces this transformation is the question the remaining sections address. The short answer is the Subjective Field—the physical mechanism that transforms coherent living brain information into feelable information, enabling access to the experiential dimension. But before turning to the clinical evidence and the field hypothesis, the next section addresses the most direct physical evidence that the experiential dimension is real: the now problem.

## Section 5: the now problem—evidence from physics

The previous sections have argued on logical and theoretical grounds that experience is an irreducible dimension. This section presents physical evidence for that claim. The evidence comes from an unexpected source: the structure of physics itself. Physics—the most precise and successful descriptive enterprise in the history of science—cannot account for the present moment. That is not a minor gap; it is a structural absence that points directly toward the experiential dimension.

### Physics has no present moment

In general relativity, proper time is geometric. It measures the length of a trajectory through spacetime—the interval along a worldline—in the same way that a ruler measures spatial distance. A clock carried along a trajectory records the length of that trajectory. It does not record a now. All events along a worldline coexist within the spacetime manifold. Proper time orders them but does not single out any event as present. The present moment is absent from the formalism entirely.

Entropy provides directionality—an arrow from past to future—but not presence. A universe with increasing entropy and no experiencing beings would have irreversible processes but no now. Things would happen in sequence, but nothing would be happening now. The arrow of time tells us which direction is later; it does not tell us which moment is present.

As Jim Holt documents in When Einstein Walked with Gödel (Holt, 2018), both Einstein and Gödel concluded that the present moment—the now—has no place in the physics of spacetime. Einstein’s special relativity showed that simultaneity is observer-dependent, eliminating any universal present. Gödel extended this using Einstein’s own equations of general relativity to argue that time as we experience it—flowing, with a genuine now—cannot be a fundamental physical reality (Gödel, 1949). The block universe contains all events coexisting with equal status. Nothing in physics singles out any moment as present. Einstein was reportedly disturbed by Gödel’s result, recognizing that it threatened to make the passage of time purely subjective. He could not refute the argument.

The now problem, then, is this: Physics describes a universe without a present moment, yet we experience a present moment. Something real exists that physics has not described.

### The now is not an illusion

The standard response is to call the now an illusion—a cognitive artifact generated by the brain’s information processing (Dennett, 1991; Frankish, 2016). If the now is not in physics, perhaps it is not real.

This response fails on its own terms. Any argument that the now is an illusion is made from within the now. The philosopher constructing the argument experiences herself as doing so in a present moment. She remembers formulating the previous sentence. She anticipates completing the next one. The now is presupposed by every act of reasoning—including the reasoning that calls it illusory. An illusion that cannot be escaped even in principle, that is presupposed by every observation and every theory, has a strong claim to being not an illusion but a datum.

Descartes recognized this structure: Whatever else can be doubted, the doubting itself cannot be. The now has the same status: It is the precondition for every observation, every measurement, every theory. It is not derived from physics: Physics is derived from observations made within it.

The now is therefore evidence. It is evidence of something real that physics has not described—a dimension whose existence physics presupposes but cannot account for. Just as planetary motion was evidence of gravity before Newton had a theory of gravity, and blackbody radiation was evidence of quantization before Planck had a theory of quanta, the now is evidence of the experiential dimension.

### The now exists only within the experiential dimension

Why does physics have no present moment? The answer the dimensional framework provides is precise: Because the present moment belongs to the experiential dimension, not to the physical dimensions.

In the lifeless block universe, all events coexist timelessly. There is no happening. There is only geometry. The now—the sense that this moment is occurring, that there is a here and a present—is what the experiential dimension adds. It is not a feature of spacetime. It is a feature of what it is like to inhabit the experiential dimension.

This is why Einstein and Gödel were right, and yet their conclusion seems paradoxical. They were right that physics has no now. The paradox dissolves when you recognize that the now was never in physics to begin with. It was always in the experiential dimension. Physics describes the block universe accurately. The experiential dimension adds the present moment to it—not by changing the physics but by adding a dimension that physics does not contain.

The now is one of the four properties that constitute the experiential dimension—alongside for-ness, within-ness, and stakes. Of these four, the now is the most directly visible to physics because it leaves a structural absence: Physics can describe everything about the universe except the one thing that every physicist experiences in every moment of doing physics. That absence is evidence. The now exists only as an experience, an experience in the experiential dimension.

### A clinical observation

I have made thousands of clinical observations over 33 years in which I am able to stimulate one brain hemisphere or the other using lateral visual field stimulation or unilateral transcranial photobiomodulation (Schiffer et al., 2021). With either maneuver, people usually exhibit reproducible and often dramatic shifts in personality, affect, and self-appraisal—qualitatively different conscious experiences from identical stimuli (Schiffer, 2000). The visual field changes induced fMRI such that the contralateral hemisphere was broadly activated (Schiffer et al., 2004). With hemispheric stimulation, my patients do not report changes in information. They report a change in how things feel—a change in the quality of their present moment experience. The anxious, threatened self that emerged from early trauma experiences the present moment differently than the calmer, more mature self. Same brain. Same information. Different experience of now (Schiffer, 2022).

This is not a philosophical argument. It is a clinical observation replicated thousands of times. The present moment—the now—is not uniform. It has qualities that differ between hemispheric selves. Those qualities cannot be accounted for by neural information processing alone. They require that their hemispheric brain information is transformed into feelable information. I have suggested that this transformation is facilitated by what I call the Subjective Field (Schiffer, 2025, 2026a)—the mechanism by which living brain information becomes feelable—and a receiving self whose developmental history shapes how the transformation is actualized.

The now problem is therefore not only a problem in physics. It is visible in the clinic. Every patient who has ever said “I feel different” after hemispheric stimulation is reporting a change in their present moment experience—a change in their access to the experiential dimension.

## Section 6: the life dimension

The previous sections have established that experience is an irreducible dimension—real, causally efficacious, and not derivable from physics. But experience does not appear everywhere. Rocks do not experience. Stars do not experience. Computers do not experience. Experience appears only in living systems—and not in all of them. Something about life is a necessary condition for access to the experiential dimension.

This section argues that life itself is an irreducible dimension—the second new dimension this paper proposes. Life is not complex chemistry. It is a categorical threshold—a phase transition into a new kind of existence. Understanding this reframes the hard problem of life in exactly the same way the dimensional argument reframes the hard problem of consciousness.

### What physics describes and what it misses

Physics and chemistry describe the mechanisms of life with extraordinary precision. We know the molecular structure of DNA, the chemistry of metabolism, the thermodynamics of cellular membranes, and the electrochemistry of neural signaling. We know how living systems maintain homeostasis, replicate, respond to their environment, and evolve. This knowledge is genuine and profound.

What we do not know—what physics and chemistry have not explained—is why any particular organization of matter crosses the threshold into living existence. Why does a cell live when its constituent molecules, separated and scattered, do not? The molecules are the same. The atoms are the same. Something is present in the living cell that is absent in its separated components—and that something is not a molecule. Immediately after a sudden death, all of the components of the live organism are present, but something is missing. It is access to the life dimension. It is not a force in the standard model. It is not a field that physics has described.

It is the life dimension.

### The origin of life problem reframed

The origin of life problem has occupied scientists for over a century. It is usually framed as a chemistry problem: Given the right molecules and the right conditions, how did the first living system assemble? Despite remarkable progress in understanding prebiotic chemistry, the threshold has not been crossed in the laboratory. We can create conditions favorable to life. We cannot create life.

I suggest this is because the origin of life problem is not a chemistry problem. It is a dimensional threshold problem. The question is not what combination of molecules produces life—it is what conditions allow physical organization to cross into the life dimension. That is a different question, and it requires a different kind of answer.

Walker and Davies (2013) argued that life has algorithmic origins—that information, not merely chemistry, is causally primary in living systems. This points in the right direction. Living systems do not merely contain information; they process it in a way that is for the organism—directed toward survival, reproduction, and maintenance. That directedness is not in the chemistry. It is a property of the life dimension.

### What the life dimension adds

What does the life dimension add that physics does not contain? Three genuinely new things.

First, goal-directedness. A living bacterium processing a chemical gradient is not merely responding to a stimulus—it is moving toward food and away from acid because that is good for it. This good-for-it is real. It is not in the chemistry of the gradient. It is a property of a system that exists within the life dimension, where information has organism-relative meaning.

Second, self-maintenance. Living systems actively maintain themselves against entropy. They consume free energy not merely to perform work but to preserve their own organization. Physics can describe conditions under which organized structures form and persist—Prigogine’s dissipative structures show that physical systems like whirlpools can sustain themselves against entropy. But dissipative structures do not repair themselves, do not replicate, and have no inside for which anything means something. The difference between a whirlpool and a living cell is not complexity. It is the categorical difference between a physical system that happens to maintain organization and a living system that exists within the life dimension.

Third, meaning-bearing information processing. A bacterium’s chemical signal is not merely a physical event—it is information that means something to the organism: approach or avoid, danger or food. This meaning is real and causally efficacious. It is what Walker and Davies called algorithmic—it operates at a level above the physical substrate. It belongs to the life dimension.

### The irreducibility of life

Life and experience are both irreducible—but in different ways. Experience is irreducible in the strongest sense: No third-person account captures what it is like to feel pain. You cannot observe experience from outside. You can only observe its physical manifestations—the tears, the cry, the behavior. These are the cross-sections of the experiential dimension intersecting with the physical dimensions, exactly as the Square in Flatland could only see the circle that was the Sphere’s cross-section in its plane. The experience itself remains inaccessible from outside.

Life is irreducible in a parallel sense. When I look at a living bacterium, I observe its movement, its chemistry, its responses. I do not observe the life itself—the aliveness that disappears at death even when all the molecules remain. Immediately after sudden death, every component is still present. The life dimension is no longer accessible. Nothing in the physical description tells you where it went—because it was never in the physical description to begin with.

Both dimensions are genuinely irreducible. Both are known through their physical manifestations without being reducible to them. The difference lies in access: Life can be confidently identified from the outside even if not explained—I can see that the bacterium is alive. Experience can only be directly known from within—I cannot feel your pain directly, only empathize with it.

### The gradient of life and experience

Not all life accesses the experiential dimension equally, or at all. This generates a gradient that the framework can describe precisely.

Bacteria inhabit the life dimension. They process meaning-bearing information and have organism-relative responses. They approach and avoid. Their information processing is for the organism in a biological sense. Whether they couple with the experiential dimension—whether there is anything it is like to be a bacterium—remains an open question. My framework does not require an answer. It requires only that the life dimension is a necessary but not sufficient condition for the experiential dimension.

Plants inhabit the life dimension. They respond to light, gravity, touch, and chemical signals.

Calvo and Friston have argued that plants engage in something resembling predictive processing (Calvo and Friston, 2017). Whether they cross the experiential threshold is uncertain. They lack the neural architecture that in animals appears to be the coupling mechanism.

Animals with nervous systems cross the second threshold—at least the more complex ones. The nervous system appears to be the biological structure associated with crossing the experiential threshold. Not all nervous systems do this equally. The richness of experience appears to scale with neural organization—not because organization produces experience, but because more organized neural systems appear better positioned to permit the transformation into feelable information.

Humans with reflective self-awareness inhabit the experiential dimension most fully—not because reflection produces experience, but because the self that receives experiential information has the most developed attractor landscape, the richest developmental history, and the greatest capacity to actualize felt experience.

### Life as gateway

The relationship between the life dimension and the experiential dimension can now be stated precisely. The biological conditions of life are necessary for the neural activity that produces brain information. It is that brain information—once transformed and received by the self—exists within the experiential dimension. The life dimension does not itself enter the experiential dimension. It is the necessary condition for the existence of the kind of information that can be transformed into feelable information. How that transformation occurs is the subject of Section 8.

The dimensional framework proposes that experiential access is universal wherever the biological coupling condition is met—across the full phylogenetic range of organized nervous systems. The biological structures supporting conscious experience in humans are not uniquely human. The limbic system, brainstem, thalamo-cortical networks, and basic architecture of neural information processing are phylogenetically ancient structures conserved across vertebrate species with remarkable genetic and architectural fidelity. The entire edifice of neuroscience research rests on this conservation—animal models of anxiety, depression, addiction, and pain are valid precisely because the neural structures supporting these experiences in mice are structurally and genetically homologous to those supporting them in humans. Conscious experience is therefore phylogenetically conserved. The difference between mouse consciousness and human consciousness is not a difference in kind but a difference in complexity and elaboration. The coupling condition is met in the mouse as it is in the human.

What differs is the richness and complexity of the brain information being transformed into feelable information by the Subjective Field.

Several species illustrate this principle with particular force. Cetaceans—dolphins and whales—demonstrate functionally identical neurological mechanisms despite radically different physical neural architectures. Dolphins practice unihemispheric slow wave sleep—one hemisphere remains fully conscious while the other sleeps—a natural demonstration of hemispheric independence of conscious experience that parallels the two-self model in a non-pathological biological context. Elephants demonstrate mirror self-recognition, multigenerational social memory, complex grief responses, and responses consistent with post-traumatic stress disorder following overwhelming experiential threat—suggesting that the troubled hemispheric self-structure may not be uniquely human but phylogenetically conserved across large-brained mammals. Cetaceans have further been shown to possess extraordinary intelligence: Dolphins possess the second highest brain-to-body mass ratio of any animal, use individualized signature whistles functioning as personal names, and process echolocation information with a sophistication that has no human equivalent. Sperm whales possess the largest brains of any animal, and recent research suggests their click communication may have combinatorial structural properties analogous to human language. African grey parrots demonstrate object permanence, numerical reasoning, and spontaneous language use. Corvids demonstrate future planning, causal reasoning, and tool use through a pallial neural architecture that lacks the layered cortical structure of mammalian brains entirely—demonstrating that the coupling condition specifies a functional biological criterion rather than a specific structural one. Colonial organisms without centralized nervous systems represent an honest boundary case—individual members with their own neural processing may have limited experiential access, but the colony as a whole does not constitute a unified experiencing system in the relevant sense. This boundary—drawn at the level of organized neural processing—is a principled and empirically grounded limit on experiential access that panpsychism cannot provide.

Several independent lines of research support this account. Feinberg and Mallatt’s analysis of the evolutionary and genetic origins of consciousness identifies the Cambrian period—approximately 520 million years ago—as the point at which organized image-forming nervous systems first met the biological conditions for experiential access, consistent with the coupling condition the dimensional framework specifies (Feinberg and Mallatt, 2013). Fabbro et al.’s examination of self- and world-consciousness across vertebrates demonstrates that self-consciousness has deep evolutionary roots across the vertebrate phylum, supporting the framework’s claim that the difference between human and non-human consciousness is one of complexity rather than categorical kind (Fabbro et al., 2015). The dimensional framework accommodates the full range of experiential states across biological diversity—including expanded states associated with meditation, flow, and eudaimonic wellbeing identified by Dresp-Langley (2022)—because these states represent variations in the character and richness of experiential access under different biological and psychological conditions, not departures from the framework’s basic account. The Force of Experience operating through pleasure, pain, meaning, and flourishing is therefore not merely a human civilizational phenomenon; it is the experiential dimension’s contribution to biological fitness across the full range of experiencing organisms, operating wherever the coupling condition is met (Di Fabio and Palazzeschi, 2015).

This is not vitalism. Vitalism proposed a special substance or force—an élan vital—present in living matter and absent from non-living matter. I am not proposing a substance. I am proposing a dimension—a threshold of organization at which matter gains access to the life dimension and acquires the properties of goal-directedness, self-maintenance, and meaning-bearing information processing. The physics is ordinary. The dimension is new.

This is not panpsychism. The experiential dimension is not present in all matter. It is accessible only through the life dimension, and the life dimension is accessible only under specific biological conditions. Most of the universe operates entirely within the physical dimensions. The experiential dimension is real but rare—confined to the small fraction of organized matter that has crossed the life threshold and then crossed the experiential threshold beyond it.

## Section 7: the subjective field—the coupling mechanism

The previous sections have established that experience is an irreducible dimension, that life is its necessary gateway, and that information processing alone cannot account for the transformation from neural activity to felt experience. Something must produce that transformation. This section proposes what that something is.

I call it the Subjective Field.

### Why a field

Physics explains transformations through fields. The electromagnetic field transforms charge into force at a distance—two magnets affect each other without touching because each exists within a field that the other perturbs. The Higgs field transforms massless particles into massive ones—without the Higgs field, the particles that make up matter would have no mass, and the universe as we know it could not exist. Fields are how physics accounts for interactions between things that do not touch in the ordinary sense, and for transformations that cannot be explained by direct mechanical contact.

The transformation from neural information to felt experience shares this character. Brain information does not mechanically produce experience. Something mediates the transformation—something that acts on coherent biological information and produces feelable information as its output. A field is the right kind of physical entity for this role.

The Subjective Field is proposed as the physical mechanism at the interface between the life dimension and the experiential dimension. It is not a field within the physical dimensions in the ordinary sense; it is the coupling mechanism by which living brain information gains access. It acts on coherent neural emissions and transforms them. The transformed information—feelable information—is what exists within the experiential dimension and can be received by the self.

A note on the use of physical-theoretic vocabulary in what follows: We invoke fields, coupling, and transformation as illustrative parallels to physical mechanisms. These analogies are conceptual rather than structural—the Subjective Field as proposed here is a conceptual placeholder for a physical mechanism whose formal specification remains future work. We do not claim that the Subjective Field has been formalized as a quantum field in the manner of the Higgs field. We claim that field-mediated qualitative transformation is a recognizable pattern in physics, that the Subjective Field is biologically necessary because experience exists and brain information is syntactic, and that formal physical specification is open work for theoretical physics in collaboration with neuroscience.

Kiyooka et al. (2026) and (Mashour, 2024) support the thesis that anesthesia works by disrupting global brain networks. When anesthesia reduces the coherence and integration of neural activity, experience diminishes or disappears. We suggest that the neural disruption under anesthesia may interfere with access to the experiential dimension, which may require coded access to the experiential dimension. The Subjective Field may transform certain coherent brain information into feelable information. We suggest that the field will not accept incoherent, segregated information.

### What the field does—the transformation

The transformation the Subjective Field performs is a type change, not a complexity increase. What changes is not the amount of information but its mode of existence—from syntactic information, which is pattern with causal structure, to feelable information, which is pattern with the potential to be experienced.

This distinction is fundamental. Syntactic information is what computers process. It has structure, causal consequences, and can be integrated, broadcast, and represented. But it has no felt character. It is not for anything. It has no stakes. It exists in the third person.

Feelable information is different in kind. It has the four properties that constitute experiential information—for-ness, within-ness, now-ness, and stakes. It is not merely processed. It is returned to the self where it can be experienced. Pain is the clearest example—pain is not simply a neural signal that reports damage and triggers avoidance behavior. Pain is felt; often it is unbearable. The unbearableness is intrinsic to the feelable information itself, not computed from it by a separate process.

The Subjective Field does not itself feel or experience. It is a transformer, not a seat of experience. This is important because it sidesteps a potential regress—critics cannot ask why the field feels, because it does not. The field transforms. The self receives. Feeling is the actualization of feelable information by a receiving self.

We do not know how the transformation occurs or the physical nature of felt information. The Higgs transformation involves symmetry-breaking, and this is a possible mechanism for the Subjective Field. What we do know is that neural information is syntactic and cannot be directly felt. Interestingly, brain surgery on an awake patient does not induce feelings when the brain is cut or manipulated. Penfield and Perot (1963) were able to stimulate the brain with electrodes and evoke experiences which felt like memories, but this may have been due to their stimulating coherent brain information that interacted with the Subjective Field.

What is clear is that we do feel and that our feelings affect our neural structures (Schiffer, 2026a). All that we feel comes from brain information, and to be felt, that brain information must undergo a transformation. We propose the Subjective Field as the mechanism by which feelings occur, by which brain information becomes feelable information which exists only in the experiential dimension. Somehow, this transformation allows feelable information to exist when the self receives it, and the self and its experiences must be within the experiential dimension. We have not discovered a particle from the field, in part because only a living organism with a functioning brain can access the experiential dimension, and living brains have never been placed in a cyclotron and likely would not function within one. Both the Subjective Field and the Experiential Dimension are appreciated out of the necessity of discovering a sensible explanation for consciousness.

Pair production in quantum electrodynamics offers a more concrete physical analog. When a high-energy photon encounters the electromagnetic field of a nucleus, the wave energy of the photon can be transformed into a particle-antiparticle pair—an electron and a positron, each with rest mass, each ontologically different from the photon that produced them. The photon is massless and travels at c; the electron and positron travel slower and possess rest mass. The transformation crosses a real categorical boundary. Crucially, this transformation cannot occur in a pure vacuum. Conservation of momentum forbids a photon traveling alone from spontaneously producing a pair. The transformation requires a third body—typically a nucleus—whose electromagnetic field provides the structure that makes the transformation possible. Wave becomes particle through field coupling.

The Subjective Field hypothesis proposes an analogous transformation in the biological domain. Coherent biophoton emissions and structured electromagnetic patterns from the living brain are wavelike—they carry information as oscillation, frequency, phase, and coherence. These wave patterns excite the electromagnetic field, where they remain photons. We propose that under conditions of sufficient coherence and structure, they also couple to a second field—the Subjective Field—and this coupling produces a different kind of excitation: experiential information with the multidimensional character of feeling. The biophoton does not become a different particle; instead it acquires an additional mode of being through coupling to an additional field.

This pattern—field-mediated transformation between qualitatively different states—is a familiar one in physics. We propose it as a conceptual model for the Subjective Field, without claiming that the Subjective Field has been formalized in the manner of these well-established fields. The Higgs mechanism, pair production, phase transitions, and superconductivity all involve fields producing qualitative change in the coupling mechanism. The Subjective Field hypothesis extends a familiar physical pattern into the biological domain, where the coupling conditions are met by living neural tissue.

### Biological confinement

Not all physical systems interact with the Subjective Field. This is biological confinement—the restriction of field coupling to living neural systems with sufficient coherence.

The principle is well established in physics. The Higgs field is omnipresent, yet the photon remains massless because it lacks the coupling parameter to interact with the Higgs boson. The strong nuclear force is fundamental, yet electrons do not participate in strong interactions because they lack color charge. Field existence does not imply universal interaction. Coupling requires the right conditions.

For the Subjective Field, the coupling conditions are biological. Only coherent, temporally organized, biologically generated electromagnetic emissions couple with the field. Random thermal noise does not couple. Simple reflexes may not couple. The organized, meaning-bearing, organism-relative neural activity of higher brain systems does couple—and the coherence of that activity determines the strength of the coupling.

This is why rocks, thermostats, and AI systems do not experience. They do not generate the coherent biological emissions that couple with the Subjective Field. The field may be present—as the Higgs field is present everywhere—but without the right coupling conditions, no transformation occurs.

The candidate physical basis for this coupling is coherent biophotonic and electromagnetic emissions from living neural tissue. Evidence exists that biophotons are produced in neural tissue (Tang et al., 2018), that myelinated nerve fibers may act as waveguides for such emissions (Rahnama et al., 2011), that biophotonic stimulation can trigger transsynaptic neural transmission (Liu et al., 2022), and that spectral properties of biophotonic emissions correlate with cognitive capacity across species (Wang et al., 2016). Whether biophotons specifically are the coupling mechanism or whether coherent electromagnetic emissions more broadly are involved remains an open question. What the framework requires is coherent biological emission—the specific physical candidate is a hypothesis for future investigation.

### The self as receiver

The Subjective Field transforms brain information into feelable information. But feelable information must be received to become actual feeling. The receiver is the self.

In this framework, the self is not a homunculus—not a little person inside the brain who watches the show. The self is the accumulated developmental history of the organism, represented neurally as an attractor landscape—a set of learned patterns, emotional associations, relational expectations, and meaning structures built up over a lifetime of experience. The attractor landscape is formed by neural structures and fields. These are dynamic, distributed networks that hold meaningful information.

When feelable information arrives, the self receives it through this attractor landscape. The attractor landscape shapes how the feelable information is actualized—which aspects are amplified, which are dampened, what emotional tone is assigned, what response is generated. The self does not produce the feeling. It actualizes the feeling—it is the structure within which feelable information becomes actual felt experience.

This is why the same feelable information can produce different experiences in different people. A piece of music that is profoundly moving to one person may leave another unmoved. An angry face that registers as a threat to a traumatized self may register as manageable to a more secure self. The transformation—what the Subjective Field does—is the same. The actualization—what the self does—differs because the attractor landscapes, the dynamic, distributed networks, differ.

It is important to clarify what is meant by the two hemispheric selves as distinct receivers of the experiential dimension. Each hemispheric self is not a localized anatomical module but a distributed network spanning multiple brain regions—with its own developmental history, attractor landscape, self-structure operator, and access to the experiential dimension. This distributed architecture makes the ontological claim more biologically plausible rather than less. Two alternative interpretations of hemispheric perceptual asymmetry warrant explicit engagement. A predictive processing account would propose that two hemispheres with different developmental histories implement different priors, producing different perceptual outputs from the same stimulus. A standard neurobiological account would invoke documented hemispheric asymmetries—right-hemisphere amygdala connectivity, negative-valence processing, left-hemisphere regulatory function—to explain different responses without requiring distinct selves. The standard neurobiological account, in its strong form, predicts that the troubled hemispheric self should reliably appear in the right hemisphere across patients. This prediction is not supported by the empirical record. Across our controlled clinical trials in which LVFS was used as a baseline measure of hemispheric emotional valence (Schiffer et al., 1999, 2020, 2021), the negative-valence hemisphere was the left hemisphere in approximately 50% of patients. The variability is further supported by the Stabell et al. (2004) Wada study of 270 epilepsy surgery candidates and our CTHEV study of 50 right-handed young adults (Schiffer et al., 2022). A theoretical synthesis developed at length in a companion case report (Schiffer, 2026a, in press) distinguishes two levels of analysis frequently conflated in this literature: lateralized emotional functions, as characterized by the emotion-type hypothesis at the population level, and hemispheric experiential dominance, as characterized by dual-brain psychology at the individual level. On this account, each hemispheric experiential system can recruit functions distributed across both hemispheres—speech, motor control, and lateralized emotional valence systems—while remaining organizationally distinct. What changes when one hemispheric self becomes dominant is not which cognitive resources are available but which experiential stance organizes the moment. The two levels are not in competition; they describe different things. The predictive processing account is similarly compatible with the dimensional framework rather than in competition with it. Predictive processing describes how each hemispheric self generates and refines brain information; the dimensional framework specifies how that brain information becomes conscious experience. The same computational mechanisms are implemented by contemporary AI systems, which show no evidence of consciousness under any reasonable criterion. Predictive processing as currently implemented is thus computationally complete without producing evident consciousness, illustrating that the framework does not reach the consciousness threshold. The argument for the dimensional interpretation does not rest on the symmetrical photograph alone but on convergent clinical evidence: Each hemispheric self exhibits a coherent and persistent attractor landscape across diverse stimuli and clinical conditions; each is addressable as a distinct interlocutor in therapy; and intervention at one hemisphere produces durable changes in self-experience rather than isolated perceptual outputs.

The self is therefore both the receiver and the agent. It receives feelable information and actualizes it as felt experience. And upon receiving it, the self becomes capable of acting—directing attention, generating thought, making decisions, recruiting motor systems. This creates the continuous loop that characterizes conscious life: Neural information is transformed into feelable information, received and actualized by the self, which then acts on the brain (Schiffer, 2026a), generating new neural information, which is transformed again (Schiffer, 2025). The loop runs continuously for as long as the organism is alive and conscious.

Mashour (2024) reviews how anesthesia disrupts consciousness through network-level changes rather than simple suppression—information is “received but not perceived”. This supports our notion that brain information must be above a threshold, coherent, and perhaps acceptable to the self.

### The formal architecture

The mathematical formalism for this framework is presented. The key elements can be stated here without mathematics.

Neural information at any moment is represented as a structured pattern of biological emissions—the brain’s electromagnetic state. The Subjective Field acts on this pattern through a transformation operator T that maps neural information into feelable information. The self acts on feelable information through a self-structure operator S that represents the organism’s accumulated developmental history. The actual experience at any moment is the result of S acting on the output of T acting on the neural information N:E = S · T · N.

This single expression captures the entire framework. N is what the brain produces. T is what the Subjective Field does. S is what the self does. E is the resulting experience.

The coupling condition specifies when T operates: only when the coherence of neural emissions exceeds a biological threshold with the correct access code. Below the threshold—in deep anesthesia, in coma, in death—no transformation occurs and E = 0. No experience. The feelable information must be acceptable to the self. Much brain information does not gain access to the Subjective Field, and some feelable information may be rejected by the self, as with repressed, traumatic, unbearable information.

The two-receiver prediction follows directly. For two hemispheric selves with different developmental histories—different operators SL, left self, and SR, right self—the same neural information N produces different experiences: EL = SL · T · N ER = SR · T · N.

Because SL ≠ SR, EL ≠ ER even when N is identical. This is the prediction the next section tests against 33 years of clinical data.

### The Wada test—one brain, one conscious self

The most direct clinical demonstration of hemispheric independence of consciousness comes from the Wada test, a procedure neurosurgeons use to lateralize language and memory before epilepsy surgery. In the Wada test, sodium amobarbital is injected into the carotid artery, temporarily anesthetizing one hemisphere while the other remains fully awake and conscious. The patient can speak on one side and use hand signals on the other to respond and report experience—but only from one hemisphere. The other hemisphere has fallen silent.

What the Wada test demonstrates is remarkable. Consciousness is not a whole-brain property. It is a property of specific neural systems that meet the conditions for coupling with the experiential dimension. When one hemisphere is pharmacologically rendered incoherent—when its organized neural activity falls below the coupling threshold—its access to the experiential dimension is lost, while the other hemisphere continues to experience normally.

This is directly relevant to Mashour’s observation that during anesthesia, sensory information is “received but not perceived” (Mashour, 2024). Neural processing continues in the anesthetized hemisphere—information arrives—but the transformation into felt experience does not occur. In the anesthetized hemisphere, the biological confinement condition of the Subjective Field has been violated: The coherent, organized emissions required for field coupling are no longer present. The information stays syntactic. It never becomes feelable.

The Wada test makes visible something my clinical work with lateral visual field stimulation suggests in a subtler form: The two hemispheres are not merely two processors of the same experience. They are two receivers with independent access to the experiential dimension. Shift which hemisphere is dominant and you shift which self is dominant in the person’s experience. Anesthetize one hemisphere completely and you silence one self entirely—while the other continues, undisturbed, in the same skull. A brain hemisphere can support a complete self, as in patients who have had one hemisphere removed by cancer surgery or stroke. At some point, less than a hemisphere will not support a mind, a conscious self.

### What we do not yet have

Intellectual honesty requires stating clearly what this framework does not provide.

It does not provide the Lagrangian of the Subjective Field—the mathematical expression that would allow physicists to calculate its behavior from first principles. It does not specify the symmetry group of the field. It does not calculate the coupling constants. These are open problems for future work—problems that require collaboration between theoretical physics and the neuroscience of consciousness.

This is not unusual in the history of physics. The Higgs field was proposed on functional grounds in 1964 and confirmed experimentally in 2012—nearly five decades between functional specification and experimental confirmation. The proposal was taken seriously not because the mathematics was complete, but because the functional specification was precise, the predictions were testable, and the framework was coherent.

A foundational objection to the Subjective Field hypothesis must be addressed directly. A reviewer might ask: The field transforms neural information into feelable information, but why does feelable information, when received by the self, produce felt experience rather than yet another syntactic process? Does the hard problem reappear inside the proposed solution?

The dimensional framework does not claim to resolve this question mechanistically—it claims to locate it precisely. The transformation from syntactical to feelable information appears to the author to be a necessary logical step: Experience exists, all experience originates in brain information, and therefore a transformation from syntactical to feelable information must occur, even if its mechanism remains unknown. The physical nature of feelable information and how brain information becomes experienced is unknown—but its necessity is logically compelling, and its existence is the most certain fact available to any experiencing being. Achieving conscious experience is foundational in exactly the sense that the existence of spatial and temporal dimensions is foundational. Physics does not explain why physical events have spatial and temporal existence—it takes dimensional existence as given and describes what occurs within dimensional boundaries. The dimensional framework makes the same move with respect to conscious experience. The Subjective Field identifies the coupling condition at which the transformation becomes possible. The self’s reception of feelable information constitutes entry into the experiential dimension as conscious experience. That this entry occurs is a foundational fact about the structure of reality. That it cannot be derived from syntactical description is precisely what the hard problem of consciousness has always shown—and what the dimensional framework acknowledges by treating consciousness as irreducible rather than claiming to reduce it. This moves the hard problem of consciousness to the same foundational level as the hard problem of space—not a solvable derivation problem but a recognition of an irreducible feature of reality.

## Section 8: clinical evidence—two selves, one brain

The previous sections established that the experiential dimension exists and that brain information must be transformed into feelable information before it can be experienced. The clinical evidence presented here does not re-establish these claims. It establishes two further things: first, that a single brain contains two distinct selves—two receivers with independent access to the experiential dimension; and second, that felt experience, once actualized within the experiential dimension, is causally real—it acts back on the brain and behavior with measurable force. These are the claims the clinical data support. These are claims that I have been positioned to make, because I have 33 years of replicated observations of two selves in one brain, controlled clinical trials, and FDA recognition of the therapeutic implications. A representative case illustrating the rapid, reversible hemispheric dissociation produced by lateral visual field viewing—including autonomic correlates and prevalence figures from a consecutive clinical series of patients—is reported separately (Schiffer, 2026b).

### The clinical observation

In 1995 I began noticing something reproducible and striking in my patients. When I asked them to limit their vision to the left or right lateral visual field (LVF)—their affect, personality, and self-appraisal would shift in ways that were often dramatic and consistent within a given patient. I tried this because of Wittling’s work, evoking different hemispheric experiences in people with an intact corpus callosum (Wittling and Roschmann, 1993). Looking into the left LVF, which preferentially activates the right hemisphere, would bring forward one emotional presentation. Looking into the right LVF, activating the left hemisphere, would bring forward another. The two presentations were qualitatively different—different in tone, different in their relationship to trauma, different in their capacity for self-compassion and mature reflection. Same brain. Same life history. Same body. Same moment. Two qualitatively different conscious experiences.

This observation has been replicated across hundreds of patients over three decades, in thousands of sessions (Schiffer, 2000, 2024) and in several placebo-controlled clinical trials (Schiffer et al., 1999, 2007, 2020, 2021). It is not subtle. Patients frequently report feeling like different people when their hemispheric dominance shifts. For example, one typical responsive patient reported while looking out his right LVF that he felt a sense of inadequacy and distress which he related to his father’s derision (Schiffer, 2000). In this state he could feel drug cravings. Looking out of the other side, he felt mature and said, “I can deal with him on this side. I know how to handle him now, and I don’t think he’d get me upset, and I wouldn’t do the counterproductive things if I looked at him out of this side all the time. I’d feel more pity for him and for the relationship, and I don’t think I’d want to abuse myself by using drugs.” The effect is reproducible, consistent within individuals, and clinically meaningful. In psychotherapy sessions, I speak separately with each presentation. About 55% of my patients are strong responders. Fifteen percent have no difference between sides.

### What the data show

The clinical observations are supported by laboratory and neuroimaging evidence.

EEG recordings and ear temperature measurements taken during lateral visual field stimulation show reliable hemispheric asymmetries—physiological differences between the two hemispheres that correlate with the reported experiential differences (Schiffer et al., 1999). The hemispheres are not doing the same thing. They are in measurably different states.

Functional MRI studies confirm that LVF stimulation preferentially activates the contralateral hemisphere—left LVF stimulation broadly activates the right hemisphere; right LVF stimulation broadly activates the left (Schiffer et al., 2004). The stimulation reaches deep into the hemisphere, not merely the visual cortex. This is consistent with the observation that the shifts involve personality and affect, not merely visual processing.

Anatomical MRI and network analyses show differences between the two hemispheres that correlate with their emotional valence—the troubled hemisphere and the more mature hemisphere differ not only functionally but structurally (Schiffer, 2022). The two selves have different neural substrates. They are not merely different modes of the same system; they are also different receivers of their recursive information.

### The clinical trials

The therapeutic implications of this framework have also been tested in placebo-controlled clinical trials using unilateral transcranial photobiomodulation—light applied to one side of the head to preferentially stimulate one hemisphere. I have studied patients with anxiety, depression, and opioid use disorder by stimulating the hemisphere associated with less distress and cravings.

The results have been striking. In a pilot placebo-controlled study of 22 participants, we found significant reductions in anxiety, depression, and opioid cravings with an effect size of 0.73 for craving reduction over placebo (Schiffer et al., 2020). We also obtained strong results in a Phase I NIH/NIDA clinical trial of 39 participants with present or past OUD and persistent cravings, treated twice a week for 4 weeks with either an active LED or sham. The active treatment reduced cravings over sham with an effect size of 1.5 and a *p*-value of 0.0001. Opioid use, mostly heroin, was also significantly reduced (Schiffer et al., 2021). These are not marginal findings; they reflect a genuine and powerful therapeutic mechanism.

In 2024, the FDA granted Breakthrough Therapy Designation to this treatment. This designation is given when preliminary clinical evidence indicates that a treatment may demonstrate substantial improvement over available therapies for a serious condition. The designation reflects the agency’s assessment that the preliminary evidence warrants priority development. The Phase II study has been completed and is awaiting FDA alignment on the primary endpoint before unblinding.

### What this evidence establishes

The clinical data establish two things directly relevant to the dimensional framework.

First, that a single brain contains two distinct selves. The hemispheres share the same blood supply, the same life history, the same body. Yet they mature at different rates, accumulate different developmental histories, and build different attractor landscapes—different neural networks that shape how each self receives and actualizes felt experience. When hemispheric dominance shifts, what changes is not merely which processor is active. What changes is which self is in the foreground—which receiver is inhabiting the experiential dimension and actualizing the feelable information that arrives there. The same stimulus, transformed by the same Subjective Field, is actualized differently by each self because each self has different information and is a different receiver. This is not two different people; it is two different selves in one brain, each with independent access to the experiential dimension.

It is worth being precise about what this establishes and what it does not. Two hemispheres with different neural substrates and different developmental histories will process information differently—this is consistent with emergence theories as well as with the dimensional framework. What the hemispheric data establish specifically is that the two selves are real, distinct, measurable, and clinically significant. They are not artifacts of stimulation. They are not two modes of one processor. They are two receivers with different histories, different attractor landscapes, and different relationships to the same event or stimulus. The dimensional framework gives this its correct description: two selves, each independently accessing the experiential dimension, each receiving feelable information through the lens of its own developmental history. One side may minimize or exaggerate a past traumatic event.

Second, that experience is causally real. If felt experience were epiphenomenal—merely what emerges from neural processing without independent causal force—then shifting hemispheric dominance would change which processor is running but would not produce the dramatic therapeutic effects we observe. Often in a therapy session, looking out of one LVF, a patient with a history of alcoholism will offer me $500 for a pretend beer in a mind experiment, but on the other side, will not take it if I try to give it away. Effect sizes of 0.73 to 1.5 for opioid craving reduction are the signature of something more fundamental than a computational shift. They reflect a change in which self is receiving feelable information within the experiential dimension—and a change in how that information is actualized. The Force of Experience is real. It acts back on the brain, on behavior, on craving, on the capacity for self-compassion and mature reflection (Schiffer, 2026a). The FDA Breakthrough Therapy Designation reflects the agency’s recognition that something real and important is happening—something that warrants priority development precisely because it cannot be explained by existing frameworks.

Experience is not what the brain produces. It is what the self receives when transformed brain information enters the experiential dimension. The clinical data show what that looks like in a real human brain—and what becomes possible when the more mature self is in the foreground.

A note on the relationship between the dimensional framework and psychoanalytic theory is warranted, given the long clinical tradition of describing two-mind phenomena in patients. Freud’s structural model identified clinically observable phenomena—primitive, reactive, fear-dominated states that produce defensive symptoms and resist rational modulation (Freud, 1923/1961). Within psychoanalytic theory, these phenomena were attributed to the id, characterized as predominantly unconscious and exerting pressure on the ego rather than directly entering conscious experience. The dimensional framework offers a different ontological account of clinically overlapping phenomena. What DBP describes as the troubled hemispheric self is fully conscious: Patients seeking therapy are consciously experiencing the depression, anxiety, addiction, or neurotic behavior that the troubled self generates, and the troubled self remains conscious and observing even when the mature self is in the foreground. In clinical work, the troubled self can be addressed, returned to, and remembers prior conversation. This is not unconscious material leaking into behavior; it is one self’s consciousness becoming the patient’s current organizing experience, with the other self continuing in the background. The ontologies of the id and of the troubled hemispheric self are therefore not equivalent. We note the clinical overlap not to claim that DBP completes or corrects psychoanalysis but to help readers familiar with psychoanalytic phenomena recognize the same clinical territory described under a different theoretical account.

A distinctive clinical capability DBP adds is selective and controlled access. Lateral visual field stimulation activates and deactivates the hemispheric experiential systems in real time, allowing the therapist to compare the two perspectives directly and engage each one in session. This methodology produces phenomena that psychoanalytic theory was not positioned to describe—rapid, reversible shifts in conscious self-state in response to manipulation of hemispheric input—and grounds the two-selves observation in a different evidential register than psychoanalytic clinical interpretation. The reference to psychoanalytic theory here is limited to its clinical observations of two-mind phenomena; psychoanalytic theory plays no role in the dimensional framework’s account of consciousness developed in the preceding sections.

## Section 9: meaning—what the experiential dimension makes possible and what its loss means

The previous sections have established that the experiential dimension exists, that life is its necessary gateway, that the Subjective Field is the coupling mechanism, and that a single brain contains two selves, each independently accessing the dimension. This section addresses what the experiential dimension makes possible—and what its loss means.

The answer is meaning itself.

### Without experience there is no meaning

A universe without the experiential dimension has geometry, causation, information, and pattern. It has nothing else. Nothing happens in it—because happening requires a present moment, and the present moment exists only within the experiential dimension. Nothing means anything in it—because meaning requires understanding, and understanding requires a being for whom information is felt rather than merely processed.

Consider the lifeless universe we described in Section 2. Space exists. Time exists. The universe expands. Black holes form. Stars collapse. Atoms interact through their forces. But nothing is occurring now. There is no now. The block universe sits complete and absolutely silent—not in the acoustic sense, but in a deeper sense: Nothing matters. No pain demands a response. No music is anything other than pressure waves. No loss is loss truly. Information exists without understanding. Pattern exists without significance.

Conscious meaning is not a property of information. It is what information becomes when it enters the experiential dimension and is received by a self. The GPS map of a city contains every street, every distance—informationally complete. But the map has no stakes. Being lost at 2 a.m. in the rain has stakes built into the experience itself. That difference is the difference between syntactic information and feelable information. It is the difference between the physical dimensions and the experiential dimension.

Understanding is not computation. A computer processing a mathematical proof does not understand it. The sense that something deep has been revealed—that a hidden structure of reality has become visible—requires a mathematician who can inhabit the experiential dimension and receive the proof there. Without the experiential dimension, there are only symbol manipulations. There is no insight.

### The dimension has valence

The experiential dimension is not neutral. This is perhaps the most important thing to say about it that has not yet been said.

Stakes are intrinsic to the experiential dimension. They cannot be turned off. Pain is unbearable not because a separate evaluative process judges it to be bad, but because unbearableness is constitutive of pain as a felt experience. The same is true of grief, of fear, of the specific ache of loss. These are not signals that report negative states. They are negative states—modes of existence within the experiential dimension that carry their weight internally.

But life is most valuable because of its positive valences—its joy, meaningfulness, positive aesthetics, experiences of love and accomplishment. There likely are other sentient life forms in the universe, but we cannot be sure of that or of what their nature might be. The human species is on the cusp of self-inflicted extinction through actions that could produce catastrophic and irreversible changes to the conditions that sustain life. If we cause our demise, the universe may revert to the silence that our little experiment on this planet tried to interrupt. We may close off our opportunities within the life and the experiential dimensions, and leave only the meaningless physical dimension—which has configurations and before and after, but not delight or the unbearable.

### Entry and exit

Entry into the experiential dimension is gradual, biological, and invisible from the inside—we find ourselves already there. No one experiences the moment of crossing the threshold into consciousness.

Exit is different. Exit is observable, irreversible, and unmistakable—and it makes the dimension more visible than any account of entry can.

Shakespeare’s Hamlet, contemplating suicide, asks whether it is nobler to suffer the slings and arrows of outrageous fortune or to take arms against a sea of troubles and by opposing end them. He is asking about the experiential dimension directly—not whether to continue existing as a physical body but whether to continue inhabiting the experiential dimension. The question only makes sense because experience has stakes. The slings and arrows are felt. They are unbearable in exactly the sense this framework describes—intrinsically demanding response, not merely signaling damage from outside. A system with no access to the experiential dimension has no slings and arrows. It processes. It does not suffer. And it has no joy to motivate it.

When a person steps off a balcony, or swallows the pills, or walks into the water—what ends is not merely neural activity. Neural activity can be reduced, disrupted, altered. What ends is the inhabiting of a dimension. The atoms remain. The neural structures persist for a brief time afterward. But the experiencing stops. Completely and immediately. Something that was present is no longer present. This is not metaphor. It is the clearest observable consequence of the dimensional framework: Exit from the experiential dimension is total, immediate, and irreversible.

Suicide and euthanasia make this most visible because they are chosen exits. A person in sufficient suffering deliberately terminates their access to the experiential dimension. This act is only coherent if the experiential dimension is real—if there is genuinely something to exit. You cannot choose to leave a dimension that does not exist. The very possibility of choosing death as a response to suffering confirms that suffering is real, that the experiential dimension is real, and that exit from it is a genuine act rather than merely the cessation of a physical process.

### Partial exits

Death is the complete exit. But the experiential dimension can also be disrupted, narrowed, or distorted without full exit.

Drug addiction is a partial and desperate attempt at exit—not from life but from the quality of experience within the experiential dimension. The person with opioid use disorder is not trying to cease neural processing. They are trying to alter or escape the felt character of their experience—to chemically modify the attractor landscape that receives feelable information, to numb the slings and arrows without leaving the dimension entirely. This is why my treatment works: not by adding a drug but by strengthening the mature self’s access to the experiential dimension so it can receive its feelable information and actualize it.

I believe psychosis attempts to disrupt the self’s capacity to accurately receive unbearable, feelable information in the experiential dimension—a different kind of partial disruption. This attempt to relieve pain, like drug abuse, fails and leads to greater dysfunction and distress.

A more common form of partial exit is the everyday clinical phenomenon of distress without understanding. Psychotherapists frequently encounter feelings of anxiety, depression, or somatic suffering not initially understood by the patient but which become meaningful through psychological work. The patient arrives with the felt experience but no narrative account of it. Therapy provides the conditions under which the experience can be received, held, and integrated. In dimensional terms, this is the recovery of experiential information that had entered the experiential dimension at the time of the original event but was pushed out of accessible experience because the receiving self at that time could not integrate it. The experience was felt; it was not held. What therapy accomplishes is not the supplying of new experience but the construction of the receiving conditions under which previously inaccessible experiential information can be brought back into integrated awareness. The therapist and the mature self of the patient together provide the scaffolding that the original receiving self lacked. This is the Force of Experience operating constructively—not as a metaphor for emotional work, but as a description of what work is being done at the level of activation barriers, attractor landscapes, and the self’s capacity to receive its own feelable information.

My clinical work has often involved people whose access to the experiential dimension has been distorted in these ways. Every patient who has said “I feel different” after hemispheric stimulation is reporting a change in their relationship to the experiential dimension.

### The stakes of the dimension

The experiential dimension is the dimension within which the universe is no longer silent. It is where information becomes understanding, where pattern becomes significant and matters, where geometry becomes presence. It is, in the end, what medicine exists to protect—not the physical substrate alone but the experiencing self that inhabits it. Psychiatry aims to improve those experiences.

## Section 10: the brain–experience Interface—observations and proposed investigations

The question of whether the experiential dimension exists does not require experimental proof. Its existence is established by direct acquaintance—every person reading this sentence knows, from the inside, that experience is real and that it has force. This is also the only way that we know of the existence of space and time. We know them only through our direct experience.

Time and space are two different dimensions that interact. Without space there would be no time, because nothing would move and no physical events would occur. Without time there could be no space, because time is necessary for movement and events. We know that spacetime creates the force of gravity. Gravity is not reducible to space or time alone—it emerges from their interaction and can only be discovered and described, not derived. Just as gravity cannot be reduced to either dimension separately but emerges from their unification, the Force of Experience cannot be reduced to biology or physics alone but emerges from the lifeexperience dimension.

Life and experience are interrelated dimensions. Experience requires life, and life is shaped by experience. Together they constitute what we call lifeexperience. We propose that the Force of Experience—the causal power of the experiential dimension acting on the physical world—is a fundamental force arising from this unified dimension, analogous in its irreducibility to gravity arising from spacetime. We propose that the Force of Experience may constitute a fifth fundamental force—one whose carrier and coupling mechanism remain to be characterized, but whose causal reality is evidenced by the entirety of human civilization.

We cannot prove life through physics, and we cannot prove experience through the physical dimension. Life happens in the physical dimension but cannot be derived from it. The same is true of experience. We can only make predictions about how the experiential dimension will act on the physical dimension—how our experiences will impact the world.

The strongest argument for the reality and causal power of the experiential dimension is not found in the laboratory, but in the accumulated material world of human civilization. Every tool ever made, every building ever constructed, every work of art ever conceived, every institution ever founded, every scientific theory ever formulated—including the theory presented in this paper—is a physical consequence of the experiential dimension acting on brains acting on matter. The experiential dimension has also created human destruction and sadism. This is the Force of Experience operating at a civilizational scale. A universe without experience would contain no human products, no science, no technology, no art—including no neuroscience and no theories of consciousness. The attempt to explain experience away using instruments and theories themselves products of experience contains a deep self-refutation.

The Force of Experience requires no exotic clinical demonstration to establish its reality. Every conscious experience contains meaningful information that acts on the brain and motivates response. The experience of threat produces fear and defensive action. The experience of love produces approach and attachment. The experience of beauty produces contemplation and creation. The experience of boredom produces seeking and restlessness. Pain and pleasure are perhaps the most fundamental and universal expressions of this force—and neither can be reduced to physicalism. The neural correlates of pain are well characterized, but nothing in that characterization explains why nociceptor activation feels bad in a way that compels avoidance. The painfulness of pain is not in the neurons—it is in the experience, and it is one of the most powerful motivating forces in nature. Every organism with a nervous system organizes its behavior around the avoidance of pain and the pursuit of pleasure. This constant and universal responsiveness to experiential content—not merely to its neural correlates but to its specific meaningful character—is the Force of Experience in its most basic and undeniable form. The accumulated product of this force across human history—every tool, building, institution, and scientific theory—is its most visible signature at the civilizational scale. The clinical evidence from hemispheric stimulation research provides its most precise measurement. But the existence of the Force of Experience does not require that measurement to be established. It requires only honest observation of what conscious experience is and what it invariably does.

The Subjective Field, we propose, is a physical field operating within the physical dimension—and as such, may in principle be discoverable as the Higgs field was discovered, once the appropriate theoretical and experimental tools exist. What the field does, however—transforming neural information into feelable experience by enabling its entry into the experiential dimension—produces consequences accessible only from within the experiential dimension itself. The field is physical. The transformation it performs crosses a dimensional boundary that no physical instrument can follow. The Subjective Field transforms neural structures and emissions into the experiential dimension by making brain information feelable—placing it inside the experiential dimension where it can be felt and known. How the Subjective Field enables this entry into the experiential dimension is as yet unknown, and may ultimately require forms of physical investigation that do not yet exist. We leave this to future explorations at the intersection of physics and biology.

This does not mean the relationship between brain and experience is beyond investigation. It means the investigations must be framed correctly. We cannot design experiments that prove or directly confirm the experiential dimension. What we can do is map the brain–experience interface—characterizing the physical events that accompany transitions in experiential intensity, the neural signatures that correlate with felt quality, and the physical consequences through which the Force of Experience leaves its mark on matter.

The proposed investigations that follow are offered in that spirit. They are not tests of the theory, but explorations of the brain–experience interface that will yield interesting results regardless of theoretical outcome—modest inquiries at the boundary of what physics and neuroscience can currently reach. They open a research program rather than close a debate.

### Proposed investigation 1: hemispheric emission asymmetry and experiential quality

Building on 33 years of hemispheric differentiation research, we propose measuring biophotonic and electromagnetic emissions from each hemisphere separately during conditions that differ in experiential quality but are matched for computational demand. If the experiential dimension acts on neural emissions in ways that reflect felt quality rather than computational load, this should be detectable as differential hemispheric asymmetry across conditions. This investigation does not directly test the Subjective Field. It asks whether experiential quality leaves a measurable physical signature—a question that is interesting regardless of theoretical outcome.

### Proposed investigation 2: the Nicolelis extension—hedonic state and syntactic transmission

Nicolelis and colleagues demonstrated that an encoder rat’s cortical activity, transmitted electronically to a decoder rat, could guide the decoder’s behavioral choices approximately 65–70% of the time. The transmitted signals carry syntactical neural information meaningful enough to guide goal-directed behavior across brains via electronic intermediary. We have proposed an extension to Dr. Nicolelis: If the encoder rat is given an opioid producing a powerful hedonic state, will the decoder rat bar-press to receive more signals from the encoder? Only syntactical content can be transmitted electronically. The experiment tests whether the syntactical content carries information sufficient to produce, in the decoder, an experience meaningful enough to motivate goal-directed behavior. No experience transfers across the wire. The Subjective Field is one fundamental field, omnipresent in the manner of gravity; what varies is coupling, with coherent biological brain information serving as what couples to the field. The framework’s claim about this experiment is that the syntactical patterns received from the encoder, when integrated into the decoder’s own coherent brain activity, may produce brain information that couples to the Subjective Field in a way that generates feelable information meaningful enough to be received by the decoder’s self and to motivate the decoder’s behavior.

The differential prediction concerns the specifics of the predicted behavioral signature. Purposeful behavior in itself does not require experience—a robot can execute syntactical commands without experiencing anything. What would be remarkable is the pattern: A decoder rat enthusiastically and repeatedly bar-pressing to receive more signals from an encoder in a known powerful hedonic state. This is not a general bar-press in response to a stimulus; it is sustained motivated approach behavior toward signals carrying information about another organism’s felt experience. The most parsimonious reading would be that the syntactical content produces, in the decoder, an experience sufficient to motivate sustained seeking—that is, that the decoder’s brain processing of the received information couples to the Subjective Field and produces experience. The dimensional framework offers one account of such a result; alternative theoretical accounts are possible, including emergentist accounts proposing that consciousness arises from sufficient signal complexity, and quantum accounts proposing that the received signals trigger experience-generating quantum events. The experiment cannot adjudicate between these interpretations directly. What it can do is establish whether the phenomenon—experience apparently arising in a decoder from electronically transmitted syntactical information about another organism’s hedonic state—is observable in living systems. If it is, the result would support theoretical accounts that have a mechanism for converting received syntactical information into experience, and would constrain accounts that do not. Two outcomes are possible. If the decoder shows no preference for signals from the euphoric encoder, the dimensional framework would need to explain why the integrated brain processing in the decoder did not couple to the Subjective Field in a manner generating motivating experience. If the decoder shows the predicted enthusiastic preference, the result would be informative across multiple theoretical frameworks—interesting on its own terms, and supportive of dimensional interpretations while not proving them. The experiment is actionable with existing infrastructure.

### Proposed investigation 3: physical correlates of transitions across the experiential threshold

Conditions that alter or eliminate conscious experience—anesthesia, psychedelics, deep meditation, hemispheric inactivation—provide natural opportunities to characterize what physically changes when experience intensifies or diminishes. We propose systematic measurement of neural coherence, biophotonic activity, and electromagnetic organization across such transitions. The goal is not to prove that consciousness causes these changes but to describe the physical landscape at the brain–experience boundary. This investigation would complement extensive existing explorations in the neuroscience of consciousness, adding the dimensional framework as an interpretive lens. Whatever the results, they will inform both neuroscience and future theoretical development.

*A note on what these investigations can establish*. None of these experiments, if positive, would prove the existence of the Subjective Field or confirm the experiential dimension directly. What they would establish is a research literature at the brain–experience interface that neither pure neuroscience nor pure philosophy currently provides. The dimensional framework generates the questions. The science that follows belongs to everyone.

## Section 11: conclusion

Two problems have resisted solution for decades because they have been framed as questions of derivation. Life and experience are not complex arrangements of what came before—they are irreducible dimensions of nature whose correct response is recognition, not derivation. Four converging lines of evidence establish this conclusion.

The AI argument provides an existence proof: Information processing of extraordinary sophistication does not generate experience, establishing that something beyond computation is required. The Now Problem provides physical evidence: The most certain datum of experience—the present moment—has no place in the formalism of physics, which is not a gap in physics but a structural absence pointing to a dimension physics does not contain. The clinical evidence provides 33 years of replicated observation that a single brain contains two selves independently accessing the experiential dimension—with therapeutic consequences sufficient to earn FDA Breakthrough Device Designation.

Together these arguments establish not merely that the hard problems are hard but that they are hard in the wrong way. The question is not how physics produces experience. The question is what conditions allow access to the experiential dimension—a tractable question that neuroscience, biology, and physics can address together.

The Subjective Field hypothesis proposes the mechanism. A physical field—potentially discoverable as the Higgs field was discovered—transforms coherent living brain information into feelable information, enabling its entry into the experiential dimension where it is received and actualized by the self. The field is physical. The transformation it performs crosses a dimensional boundary. The self that receives feelable information is not a homunculus but an attractor landscape built from developmental history—which is why the same external stimulus produces different experiences in different people, and why the same brain produces different experiences from each hemisphere.

Life and experience together constitute what we call lifeexperience—a unified dimension from which emerges the Force of Experience, which we propose as a candidate fifth fundamental force. Its carrier and coupling mechanism remain to be characterized. Its causal reality needs no laboratory confirmation. Every instrument ever built to study the brain—every fMRI scanner, every electrode, every cyclotron—is itself a product of the Force of Experience acting on matter. The attempt to explain experience away with instruments that experience built contains a self-refutation that no future data can resolve.

Many open problems remain—formal physical specification of the Subjective Field, identification of the biological conditions that determine coupling strength, and characterization of the Force of Experience including its carrier, coupling mechanism, and the precise relationship between experiential content and physical action. These are problems for physicists, neuroscientists, and philosophers working together—problems that become tractable only once the dimensional framework is accepted as the correct starting point.

The hard problems are not solved by finding a better mechanism. They are addressed by the recognition that dimensions do not require mechanisms for their existence.

Space exists. Time exists. Life exists. Experience exists. These are not conclusions; they are the beginning of real science—the recognition from which everything else can be built.

## Data Availability

The original contributions presented in the study are included in the article/supplementary material, further inquiries can be directed to the corresponding author.
